# PARP inhibition in breast cancer: progress made and future hopes

**DOI:** 10.1038/s41523-022-00411-3

**Published:** 2022-04-08

**Authors:** Nadine Tung, Judy E. Garber

**Affiliations:** 1 Beth Israel Deaconess Medical Center, Boston, MA USA; 2 Harvard Medical School, Boston, MA USA; 3 Dana-Farber Cancer Institute, Boston, MA USA

**Keywords:** Oncology, Breast cancer

## Abstract

PARP inhibitors have been approved for the treatment of metastatic breast cancer in germline *BRCA* mutation (g*BRCA*m) carriers. The recent OlympiA trial demonstrated improved progression-free and distant disease-free survival with adjuvant olaparib for g*BRCA*m carriers with HER2-negative high-risk early-stage breast cancer. The current article addresses some for the questions raised by OlympiA regarding how to incorporate PARP inhibitors into the treatment of early-stage breast cancer as well as future directions for PARP inhibitors in breast cancer treatment and prevention.

The publication of the OlympiA trial^1^ provides an opportunity to reflect on the progress made with PARP inhibitors in the treatment of breast cancer and to contemplate what the future might hold for them. With PARP inhibitors approved for germline *BRCA* mutation (g*BRCA*m) carriers in the metastatic setting and recently approved for early-stage disease, it is worth considering how the efficacy of PARP inhibitors might be improved and how the population of patients with breast cancer who could benefit from PARP inhibitors could expand beyond g*BRCA*m carriers (the term mutation will be used to indicate pathogenic or likely pathogenic gene variants).

Four PARP inhibitors are now approved for the treatment of four *BRCA*-associated cancers, namely ovarian, pancreatic, prostate, and breast cancer. In ovarian cancer, PARP inhibitors are approved to treat recurrent cancer and as maintenance therapy after chemotherapy in those with platinum-sensitive disease^2–8^. In pancreatic cancer, PARP inhibitors are also approved as maintenance therapy in g*BRCA*m carriers with metastatic disease that does not progress after platinum chemotherapy^9^. Moreover, in castrate-resistant prostate cancer, they are approved for treatment in patients with *BRCA* mutations, or mutations in other homologous recombination (HR)-related genes, though the efficacy in those without a *BRCA* mutation is less clear^10^.

In breast cancer, two PARP inhibitors, olaparib and talazoparib, have been approved for treatment of g*BRCA*m carriers with metastatic HER2-negative breast cancer based on the OlympiAD and EMBRACA trials, respectively^11,12^. These phase 3 trials compared single-agent olaparib or talazoparib to non-platinum single-agent chemotherapy in g*BRCA*m carriers with metastatic disease. Both studies demonstrated that, compared with chemotherapy, the PARP inhibitor resulted in a significant improvement in progression-free survival (PFS) and health-related quality of life, though not overall survival. The response rate with both PARP inhibitors was ~60% and the median duration of response was ~6 months. These results led to regulatory approval and guideline recommendations to offer any patient with metastatic HER2-negative breast cancer the opportunity to have germline genetic testing if a PARP inhibitor would be used for treatment.

The OlympiA trial was a large international randomized trial that evaluated 1 year of adjuvant olaparib vs. placebo after chemotherapy and local treatment in g*BRCA*m carriers with HER2-negative breast cancer and a high risk of recurrence^1^ (Fig. 1). Eligibility included g*BRCA*m carriers who had residual disease after at least six cycles of neoadjuvant chemotherapy (95% of whom received anthracycline/alkylator/taxane-based chemotherapy), with a higher tumor burden required for those with hormone receptor-positive disease (i.e., clinical stage, pathologic stage, estrogen receptor and tumor grade (CSP + EG) score ≥ 3). For those who underwent surgery first, patients with triple-negative breast cancer (TNBC) must have had a tumor larger than 2 cm or axillary involvement, and those with hormone recptor-positive disease needed at least four involved axillary nodes. Based on an event-driven interim analysis, the trial was stopped early because superiority was observed for the olaparib arm. Olaparib resulted in a significantly better invasive disease-free survival (iDFS) [hazard ratio 0.58; *p* < 0.0001] and distant disease-free survival (DDFS) (hazard ratio 0.57; *p* < 0.0001) with a decrease in distant recurrences driving results. While overall survival was numerically better with olaparib, the stringent requirement for significance was not met at the initial analysis. With a significant decrease in distant recurrences, there was the expectation that olaparib might eventually result in a survival benefit. In March 2022, a significant overall survival benefit was reported at the virtual plenary session of ESMO (hazard ratio of 0.68; *p* = 0.009).

**Fig. 1:**
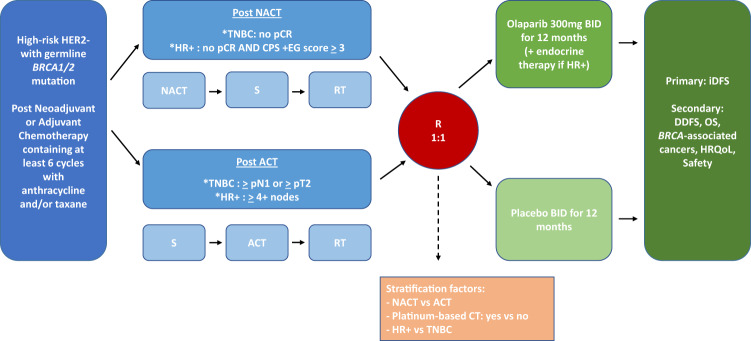
Schema of the OlympiA trial. NACT neoadjuvant chemotherapy, ACT adjuvant chemotherapy, S surgery, RT radiation therapy, HR+ hormone receptor-positive, TNBC triple-negative breast cancer, R randomized, iDFS invasive disease-free survival, DDFS distant disease-free survival, OS overall survival, HRQoL health-related quality of life, CT chemotherapy.

The practice-changing results of OlympiA underscore the importance of identifying g*BRCA*m carriers among patients with early-stage breast cancer who can benefit from a PARP inhibitor in addition to those with advanced disease. While germline testing in the early-stage setting had previously been used primarily for surgical decisions and subsequent surveillance, this information is now also needed for systemic treatment decisions. With recent data about the frequency of mutations in unselected patients with breast cancer, including older patients^13,14^, it may be more useful to identify which patients do NOT need testing, (e.g., older patients with hormone receptor-positive breast cancer and no family history of *BRCA*-related cancers) rather than complicated algorithms for which patients do. Alternatively, as was the case for patients with metastatic HER2-negative breast cancer, any newly diagnosed breast cancer patient who meets eligibility for OlympiA should be offered germline testing to identify those who might benefit from olaparib. Unfortunately, germline testing is offered to fewer than half of patients with breast cancer who meet criteria for testing and underutilization is greatest among underserved populations^15–19^. Some may reasonably argue that the time for universal germline testing has arrived for patients with breast cancer. Regardless, alternative genetic testing models are needed urgently, such as oncology-led germline testing for cancer patients, with standardized ways of providing essential pre-test education, in order to meet the increasing need for testing.

Despite the exciting results of OlympiA, several questions remain regarding the use of olaparib in g*BRCA*m with early-stage breast cancer. Is there a way to apply the results of OlympiA to a larger population of g*BRCA*m carriers with hormone receptor-positive disease than were included in the trial? In OlympiA, the bar for eligibility was set higher for patients with hormone recptor-positive disease, with at least four involved nodes if adjuvant or CBS + EG score 3 or higher if neoadjuvant. This resulted in a particularly high-risk hormone receptor-positive cohort with 3 year iDFS of 77% in the placebo arm, which is much higher than in many other trials of hormone receptor-positive disease. This begs the question as to whether olaparib might also benefit g*BRCA*m carriers with lower volume hormone recptor-positive disease such as those with fewer involved axillary nodes. It seems unlikely that clinical trials specifically for this subgroup of patients will be forthcoming. Another way to expand the number of carriers with hormone receptor-positive disease who might benefit from olaparib pertains to those whose tumors have low estrogen receptor (ER) expression. In OlympiA, ER and progesterone receptor (PR) expression greater than 1% was considered hormone receptor-positive. However, other data suggest that tumors with <10% ER expression have morphologic and molecular features as well as chemotherapy responsiveness more like TNBC than the usual type of ER-positive breast cancer^20–22^. Thus, for tumors with <10% ER expression, it is tempting to consider using the OlympiA eligibility criteria for TNBC rather than those for hormone receptor-positive disease, thereby allowing more patients with some ER expression to benefit from olaparib.

Another question relates to the optimal duration of adjuvant olaparib in g*BRCA*m carriers with early-stage breast cancer. In the ovarian cancer trials (e.g., SOLO-1, PRMIA)^2,7^ PARP inhibitors were employed for at least 2 years or until progression, compared to the 1 year of adjuvant olaparib in OlympiA. However, patients with ovarian cancer have a much higher risk for relapse, and longer PARP inhibitor use could result in a higher rate of myelodysplastic syndrome (MDS) or acute myelogenous leukemia (AML). More is not always better, as demonstrated by the HERA trial that failed to show superiority for 2 years of adjuvant trastuzumab compared to 1 year^23^. An important question relates to the safety of PARP inhibitors. While it is reassuring that no increase in MDS, AML or other serious adverse events such as pneumonitis or non-breast primary cancers were observed with olaparib in OlympiA, continued follow-up is essential.

Questions also arise about the optimal way to integrate olaparib with other adjuvant therapies that were not standard when OlympiA was designed, such as a CDK4/6 inhibitor for those with hormone receptor-positive disease and immune therapy for those with TNBC. There are no data directly comparing a CDK4/6 inhibitor to a PARP inhibitor in g*BRCA*m carriers with breast cancer. Given the negative results from the PALLAS^24^ and Penelope-B^25^ adjuvant trials and the desire by many to ensure that the positive results of monarchE are maintained with longer follow-up^26^, olaparib provides a good option for appropriate g*BRCA*m carriers with hormone receptor-positive disease. Since eligibility for monarchE included some patients with 1–3 involved nodes, g*BRCA*m carriers unable to meet criteria for olaparib may fulfill eligibility for adjuvant abemaciclib. There are no safety data for combining a PARP inhibitor and a CDK4/6 inhibitor for patients with early stage breast cancer, and this is not recommended. One must also consider the possibility of antagonism between the two agents if administered simultaneously, since CDK4/6 inhibitors will stall cells at the G1/S transition in the cell cycle, preventing S phase entry, which is critical for PARP inhibitor cytotoxicity.

What about adjuvant capecitabine in g*BRCA*m carriers with TNBC? Adjuvant capecitabine for patients with triple-negative breast cancer who had residual disease after neoadjuvant chemotherapy was not permitted in OlympiA. However, in the OlympiAD and EMBRACA trials, single-agent PARP inhibitor was superior to single-agent chemotherapy in the metastatic setting: capecitabine was received by 45% of patients in the chemotherapy arms of both trials^11,12^. In addition, an analysis of patients from OlympiAD treated in the first line metastatic setting demonstrated a survival benefit with olaparib suggesting that earlier PARP inhibitor use is beneficial^27^. Thus, while there are no data comparing adjuvant capecitabine to olaparib, there are data to suggest that olaparib may be the better choice for g*BRCA* carriers with TNBC. Of note, as there are no safety data for combining capecitabine with olaparib and this should be avoided outside of a trial.

Should olaparib be combined with a PD-1 or PD-L1 inhibitor in g*BRCA*m carriers with TNBC? Data from the KEYNOTE-522 and GeparNuevo trials demonstrate that outcomes are superior for patients with clinical stage II-III TNBC who receive immune checkpoint blockade (ICB) plus chemotherapy. For g*BRCA*m carriers with TNBC, should PD-1/PD-L1 blockers and olaparib be administered together in the adjuvant setting? We learned from IMpassion130 that among TNBC, tumors in g*BRCA*m carriers do not express PD-L1 more often and g*BRCA*m carriers do not appear to receive more benefit from atezolizumab than non-carriers in the metastatic setting^28^. Both the MEDIOLA and TOPACIO (Keynote 162) trials have reported activity combining a PARP inhibitor with immune therapy for patients with *BRCA* mutations and metastatic breast cancer, but neither had a comparator arm with a PARP inhibitor alone^29,30^. Several ongoing trials are evaluating combinations of a PARP inhibitor and ICB. Given the 60% response rate of a PARP inhibitor alone in OlympiAD and EMBRACA, as well as the cost and potential toxicity of ICB, data demonstrating superiority for the combination compared with single-agent PARP inhibitor in g*BRCA*m carriers would be important. Yet, for those with TNBC in OlympiA, 14% still had an invasive recurrence and 12.5% a distant recurrence with olaparib after intensive chemotherapy^1^, leaving ample room for improvement in outcomes that might be provided by the addition of immunotherapy. However, there is not yet a biomarker that predicts benefit for the addition of ICB to a PARP inhibitor. The challenge will be ultimately discriminating which g*BRCA*m carriers need ICB in addition to olaparib from those cured without the additional toxicity and cost of immune therapy. Until there are more data, the use of adjuvant immune therapy in combination with olaparib will have to be individualized based on the pathologic response to neoadjuvant therapy, the initial clinical stage, and tolerance to therapy. For g*BRCA*m carriers with TNBC and residual disease after neoadjuvant chemotherapy, or who undergo surgery first and have large tumor size or nodal involvement, the addition of immune therapy to olaparib is a reasonable option.

Many have questioned whether adding a PARP inhibitor to chemotherapy would improve efficacy for g*BRCA*m carriers. It has not been easy to combine PARP inhibitors with cytotoxic chemotherapies due to overlapping myelosuppression. It is also not clear that these combinations are more effective than chemotherapy alone. In the neoadjuvant setting, the BrighTNess trial did not find that the addition of veliparib increased pCR rate when added to taxane/platinum plus anthracycline-based chemotherapy^31^. In the metastatic setting, the addition of veliparib to paclitaxel and carboplatin significantly improved median PFS by 2 months in the randomized BROCADE3 trial^32^. However, since patients were allowed to continue veliparib (or placebo) after chemotherapy was stopped and the PFS curves appeared to separate after most patients stopped chemotherapy, one might ask whether the benefit of veliparib reflects maintenance use of a PARP inhibitor after chemotherapy rather than benefit from the combination of chemotherapy with a PARP inhibitor. However, while veliparib inhibits the catalytic activity of PARP1 well, it has less potency for PARP1 trapping compared with other PARP inhibitors. This may facilitate combination of veliparib with chemotherapy, but also suggests that results might differ with other PARP inhibitors^33^. The neoadjuvant GeparOLA trial suggests that platinum can be replaced by olaparib during chemotherapy for g*BRCA*m carriers with breast cancer. In that trial, comparable pCR rates were seen with paclitaxel/olaparib and taxol/carboplatin in patients with germline or somatic *BRCA* mutations or a high homologous recombination deficiency (HRD) score^34^. The question of whether a PARP inhibitor is just another form of cytototoxic chemotherapy remains unanswered at this time.

Is there a role for using PARP inhibitors in g*BRCA*m carriers with breast cancer in the neoadjuvant setting? OlympiA demonstrated that adding a PARP inhibitor to standard anthracycline/alkylator/taxane-based chemotherapy improves iDFS and DDFS in g*BRCA*m carriers with a high risk of recurrence. However might the neoadjuvant setting allow de-escalation or even omission of chemotherapy for some g*BRCA*m carriers, especially those with lower risk breast cancer who might have a pathologic complete response (pCR) with a PARP inhibitor alone? Use of neoadjuvant talazoparib resulted in a 53% pCR rate in a small study of g*BRCA*m carriers, most of whom had TNBC^35^. In NeoTALA, the larger expansion trial in g*BRCA*m carriers with TNBC only, pCR rate was 46% in the 48 mutation carriers who were evaluable for response^36^. With data that a pCR achieved with less intense chemotherapy may have the same excellent outcomes as pCR attained with more chemotherapy^37^, this approach is being evaluated in patients with HER2+ breast cancer (CompassHER2 pCR; NCT04266249) and merits investigation in g*BRCA*m carriers.

There is a critical need to understand primary and acquired resistance to PARP inhibitors since at least half of g*BRCA*m carriers do not respond to PARP inhibitors in the neoadjuvant setting^35,36^ and the median duration of response is only six months in the metastatic setting^11,12^. There are various potential mechanisms of resistance which are being evaluated. We eagerly await the results of the translational analyses planned from the OlympiA trial and hope they will shed light on predictors of response and resistance to PARP inhibitors. Broadly speaking, *BRCA*-deficient tumor cells can potentially become resistant to PARP inhibitors by restoring homologous recombination (HR) repair or by stabilizing their replication forks. HR function can be restored through *BRCA* reversion mutations^38,39^, and potentially through loss of 53BP1, which inhibits homologous recombination^40,41^, or through de-methylation of the *BRCA*1 promoter^42^. In addition, an increase in the P glycoprotein efflux pump may remove the PARPi from the cancer cell^43,44^. Changes in the PARP1 protein may also cause PARP inhibitor resistance, such as mutations to the DNA binding site^45^ or changes that restore its catalytic activity^46^. In *BRCA*1 mutation carriers only, mutations in non-homologous end joining increase DNA repair through an HR-like pathway that is independent of *BRCA*1, but requires intact *BRCA*2^47^. Loss of the PARP1 protein is another potential mechanism of PARP inhibitor resistance. And finally BRCA1, BRCA2, and PARP1 protect stalled replication forks allowing fork repair after DNA damage. If the replication fork is not repaired and collapses, that leads to cell death. Mechanisms that stabilize the replication fork may potentially lead to PARP inhibitor resistance^48–51^. Many ongoing trials are evaluating PARP inhibitors combined with other agents specifically targeted to overcome resistance, including combination with inhibitors of *ATM*/ ATR, Chk1, WEE1, VEG-F, Hsp90, PI3K, MEK, BET/BRD4, CDK12, Pol Θ, and MMEJ, among others. Another promising approach to overcome resistance includes targeting immunosuppressive macrophages in the tumor^52^.

Despite the excitement from the results of OlympiA, no more than 5% of all patients with breast cancer have a g*BRCA*m^13,14^. Can we identify patients with breast cancer who do not have a g*BRCA*m but might also benefit from PARP inhibitors? TBCRC 048 was a phase 2 trial that evaluated olaparib in patients with metastatic breast cancer and either a germline mutation in an HR-related gene other than *BRCA1/2* or a somatic mutation in an HR-related gene, including *BRCA1/2*, if a germline *BRCA* mutation was absent^53^. Among patients with a g*PALB2* mutation, 82% had a response and all had clinical benefit. Fifty percent of the patients with a somatic *BRCA* mutation also had a response and two-thirds had clinical benefit. Yet no patients with only an *ATM* or *CHEK2* mutation responded, demonstrating that the particular HR-related gene mutated matters. Since 87% of the 54 patients in this trial had a mutation in *BRCA1/2*, *ATM*, *PALB2* or *CHEK2*, there were insufficient data to comment on mutations in other HR-pathway genes. Of note, most patients enrolled and therefore most responses observed were in patients with estrogen receptor (ER)-positive breast cancer, again underscoring the importance of not limiting evaluation of PARP inhibitors to TNBC. In addition, it is worth remembering that 77% of the breast cancers that develop in g*BRCA2*m carriers are ER-positive. Thus, the majority of g*BRCA2*m carriers who responded in OlympiA likely had an ER-positive breast cancer.

How else might breast cancers with HRD enabling PARP inhibitor response be identified? In addition to testing for mutations or promoter methylation in HR-related genes, functional assays may be useful^54^, or genomic scars, such as copy number variations in the tumor. Alternatively, whole exome or genome sequencing (e.g., HRDetect) can be used to detect tumor mutational signatures characteristic of those found in cancers from g*BRCA*m carriers. Using HRDetect, investigators report that nearly 60% of early-stage TNBC have HRD^55^. Several studies have evaluated the benefit of a PARPi in patients without a g*BRCA*m who have breast cancers with HRD, most often TNBC, and responses have been seen^34,56–58^. Despite progress, we still await the optimal assay to reliably measure HRD. One limitation of these predictive biomarkers (other than functional assays) is that they represent genomic scars or patterns that existed at some point, but may not indicate extant HR deficiency, needed for PARP inhibitor response. In addition, predicting PARP inhibitor response in patients without a germline *BRCA* mutation may not simply rely on identifying HRD in a tumor. And finally, now that PARP inhibitors are approved for g*BRCA*m carriers with metastatic breast cancer and early-stage breast cancer, is it time to tackle the last frontier of prevention? How might PARP inhibitors be used to significantly reduce the risk of breast, ovarian and other *BRCA*-related cancers in gBRCAm carriers? Several challenges exist before a large prevention trial can be undertaken. Since risk is a lifelong challenge, evaluation of the lowest effective PARP inhibitor dose, different drug schedules including intermittent exposure, and potential modifiable intermediate biomarkers would be important areas for investigation. For example, would intermittent exposure be effective or merely select for resistance to PARP inhibitors and other therapies targeting this pathway? It will be interesting to observe the incidence of second breast, fallopian tube/ovarian and other cancers in both arms of the OlympiA trial to see if olaparib leads to a decrease in these cancers. Of course, since 75% of patients in OlympiA had bilateral mastectomies, assessing any decrease in contralateral breast cancer with olaparib will be limited.

After years of phase 2 trials with PARP inhibitors in metastatic breast cancer, when OlympiAD and EMBRACA were published, some commented that we were at the end of the beginning of the PARP inhibitor journey in breast cancer. Now, with the enormous success of OlympiA, we are certainly well into the middle. Eliminating the need for chemotherapy for more g*BRCA*m carriers with breast cancer, expanding the population of patients with breast cancer who can benefit from a PARP inhibitor and reducing cancer risk for g*BRCA*m carriers so that prophylactic surgeries are no longer needed are among the dreams of the future. Only then will we arrive at the end of the journey.

## Reporting summary

Further information on research design is available in the Nature Research Reporting Summary linked to this article.

## Supplementary information

Reporting Summary Checklist
